# Functional Evaluation of a Novel Homozygous ADCY3 Variant Causing Childhood Obesity

**DOI:** 10.3390/ijms252111815

**Published:** 2024-11-03

**Authors:** Idris Mohammed, Senthil Selvaraj, Wesam S. Ahmed, Tara Al-Barazenji, Hajar Dauleh, Donald R. Love, Luis R. Saraiva, Khalid Hussain

**Affiliations:** 1 College of Health & Life Sciences, Hamad Bin Khalifa University, Doha P.O. Box 34110, Qatar; 2 Division of Endocrinology, Department of Pediatric Medicine, Sidra Medicine, Doha P.O. Box 26999, Qatar; 3 Division of Translational Medicine, Research Branch, Sidra Medicine, Doha P.O. Box 26999, Qatar; 4 Division of Genetic Pathology, Department of Pathology, Sidra Medicine, Doha P.O. Box 26999, Qatar; dlove@sidra.org; 5 Department of Comparative Medicine, Yale University School of Medicine, New Haven, CT 06520-8016, USA

**Keywords:** ADCY3, monogenic obesity, childhood obesity, adenylate cyclase-3

## Abstract

Adenylate cyclase 3 (*ADCY3*) is a transmembrane protein predominantly expressed in the primary cilia of neurons. It plays a vital role in converting ATP to cAMP, a secondary messenger that regulates various downstream signaling pathways such as carbohydrates and lipids metabolism. Homozygous loss-of-function variants in the *ADCY3* gene lead to severe early-onset obesity and insulin resistance whereas gain-of-function variants protect against obesity. To describe a novel pathogenic *ADCY3* variant implicated in early-onset obesity and functionally characterize this variant via in vitro and in silico validation, we identified a novel homozygous nonsense variant c.2520C>G, p.Thr840X in the *ADCY3* gene using gene panel sequencing in a four-year-old girl. She was born to first-cousin consanguineous parents. The patient presented with severe obesity, and exhibited hepatomegaly and insulin resistance, with other biochemical and hormonal tests being normal. In vitro and in silico functional analyses showed downregulation and impaired activation of the ADCY3 protein. Our findings contribute to existing research that supports the role of *ADCY3* in the genetic pathogenesis of early-onset obesity. In vitro and in silico functional characterization of the novel p.Thr840X variant showed impaired enzymatic activity leading to receptor loss of function, consistent with the patient’s phenotype. Genetic testing is essential in severe early-onset obesity and early diagnosis could benefit patients with personalized treatment strategies.

## 1. Introduction

Monogenic obesity is severe early-onset obesity due to a single gene in the leptin–melanocortin signaling pathway. Most pathogenic variants in this pathway follow an autosomal recessive pattern of inheritance [1]. Genome-wide association studies (GWAS) have facilitated the discovery of genes that contribute to monogenic obesity, one of which is adenylyl cyclase 3 (*ADCY3*), a member of cyclases significantly associated with obesity [2]. *ADCY3* is a member of the adenylyl cyclase enzyme family that catalyzes cyclic adenosine monophosphate (cAMP), a secondary messenger from adenosine triphosphate (ATP), in response to G-protein coupled receptors (GPCRs) binding. *ADCY3* encodes a transmembrane protein located on the short arm of chromosome 2, 2p23.3, with 21 exons and localizes in the primary neural cilia of the hypothalamus [3].

Studies in mice and humans show strong evidence that *ADCY3* is vital in moderating energy homeostasis [4,5]. One study showed that homozygous *ADCY3* knock-out mice are obese, hyperphagic, and have leptin resistance [6]. In contrast, in another study in mice, a gain-of-function mutation in the *ADCY3* had a protective effect against diet-induced obesity [7]. Several studies in humans also confirm the association of *ADCY3* polymorphisms with obesity. For instance, homozygous *ADCY3* pathogenic variants lead to severe obesity in the Pakistani population [5], a Swedish study of 630 men found that variants in the *ADCY3* gene confer risk to obesity [8], and some *ADCY3* variants are associated with adult-onset obesity in the Chinese Han population [9].

Recently, a study found that ADCY3 colocalizes with the MC4R gene, the most common cause of monogenic obesity, at the neural primary cilia in the hypothalamic neurons [10]. This subcellular localization could lead to impaired signaling pathways in neuronal primary cilia, affecting appetite regulation and energy homeostasis, and could be a genetic predisposition for ciliopathies associated with childhood obesity [11].

In this study, we describe a four-year-old female child born to consanguineous Pakistani parents who presented with early-onset obesity and signs of insulin resistance. Genetic testing revealed a novel homozygous c.2520C>G p.Thr840X variant in the *ADCY3* gene, which was in a heterozygous state in both parents. We performed clinical, genetic, and biochemical investigations to assess the pathogenicity of this novel variant.

## 2. Results

### 2.1. Case Presentation

A 4-year-old female Pakistani child was born to first-cousin parents at term with a birth weight of 3.5 kg. The patient started to gain weight when she was one year old. Her weight at the time of recruitment when she was two years old was 26 kg (100th percentile), with a BMI of 27.4 kg/m^2^ +4.63 SD. She had hyperphagia and signs of insulin resistance (acanthosis nigricans on the neck and flexural areas). Her liver ultrasound showed a mild degree of hepatomegaly. Her mother was overweight, with a BMI of 29 kg/m^2^, and her father was obese, with a BMI of 37 kg/m^2^, and has a strong family history of obesity and type 2 diabetes (T2D).

### 2.2. Genetic Analysis

Sequencing a customized gene panel of 52 obesity-related genes, we identified a novel homozygous nonsense variant, c.2520C>G p.Thr840X, in the *ADCY3* gene. This nonsense variant is located in the transmembrane region of the receptor and is predicted to lead to a premature stop codon at position p.Thr840X. This novel variant is predicted to have a deleterious effect on the receptor according to several in silico prediction tools (MutationTaster version 2021, CADD). The variant is located in a region highly conserved between several species. The variant was not found in the Genome Aggregation Database (gnomAD) “https://gnomad.broadinstitute.org/ (accessed on 28 August 2024)” and its Combined Annotation Dependent Depletion (CADD) “https://cadd.gs.washington.edu/snv (accessed on 28 August 2024)” score is predicted to be 47, suggesting the variant to be pathogenic. According to the clinical interpretation of genetic variants by The American College of Medical Genetics and Genomics (ACMG), the variant is predicted to be PM2/PP3/PVS1, which is interpreted as pathogenic.

### 2.3. Quantitative Analysis of CRE and SRE Luciferase Activity and cAMP Levels in 3T3-L1 Cells Expressing Wild-Type and Mutant ADCY3

**Figure 1:**
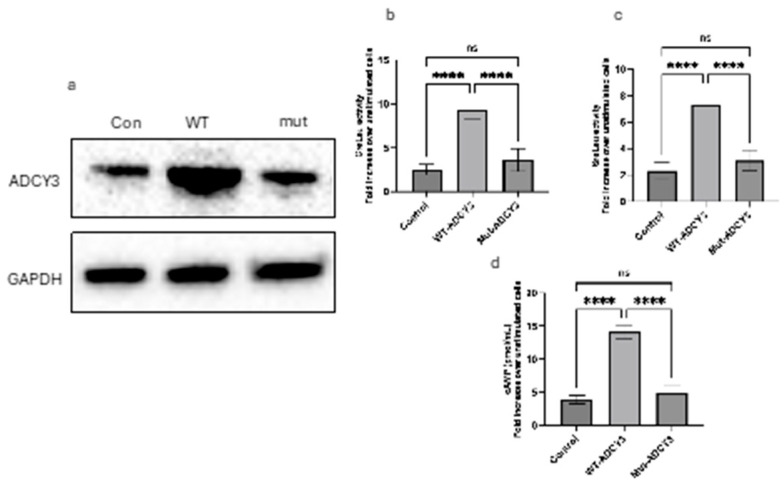
Impact of wild-type and mutant ADCY3 on protein expression, signaling pathways, and cAMP production in 3T3-L1 cells. (**a**) Western blot showing ADCY3 protein levels in 3T3-L1 cells transfected with wild-type (WT) or mutant ADCY3. GAPDH was used as a loading control. (**b**) CRE-luciferase activity in 3T3-L1 cells expressing WT or mutant ADCY3. Cells were treated with forskolin, resulting in a 2.5-fold increase in CRE-luciferase activity in WT ADCY3-expressing cells compared to unstimulated controls, while mutant ADCY3 showed significantly reduced activity (Control: 2.5 ± 0.61; WT-ADCY3: 9.33 ± 1.11; Mut-ADCY3: 3.62 ± 1.27; n = 3). (**c**) SRE-luciferase activity in 3T3-L1 cells expressing WT or mutant ADCY3. Forskolin treatment led to a nearly 3-fold increase in SRE-luciferase activity in WT ADCY3-expressing cells, whereas mutant ADCY3 showed markedly lower activity (Control: 2.32 ± 0.65; WT-ADCY3: 7.28 ± 1.13; Mut-ADCY3: 3.1 ± 0.76, n = 3). (**d**) Intracellular cAMP levels in 3T3-L1 cells expressing WT or mutant ADCY3 following forskolin treatment. WT ADCY3 significantly increased cAMP levels, while mutant ADCY3 exhibited only a modest increase (Control: 3.89 ± 0.63; WT-ADCY3: 14.10 ± 1.01; Mut-ADCY3: 4.84 ± 1.22, n = 3). Error bars represent the standard deviation (SD) from three independent experiments. **** *p* < 0.0001. Note: ns: not significant.

To elucidate the functional impact of wild-type (WT) and mutant ADCY3 on cAMP-mediated and MAPK/ERK-mediated transcriptional activities, we conducted CRE and SRE luciferase reporter assays, along with cAMP quantification, in 3T3-L1 cells. Cells were transiently transfected with either WT or mutant ADCY3, along with the appropriate luciferase reporter constructs. Following transfection, cells were treated with forskolin for 4 h to stimulate the respective signaling pathways, and luciferase activity and cAMP levels were subsequently measured (Figure 1a).

### 2.4. CRE-Luciferase Activity

A significant difference in CRE-luciferase activity was observed between cells expressing WT and mutant ADCY3 when normalized to their respective unstimulated controls. WT ADCY3-expressing cells exhibited a pronounced increase in luciferase activity, showing an approximate 2.5-fold enhancement relative to unstimulated WT cells. This robust elevation reflects effective cAMP production and subsequent CRE-mediated transcriptional activation, consistent with the known role of ADCY3 in cAMP signaling. In contrast, cells expressing the mutant ADCY3 showed a markedly reduced CRE-luciferase activity, with only a slight increase compared to their unstimulated counterparts, indicating a significant impairment in the mutant ADCY3’s ability to facilitate cAMP production and downstream signaling (Figure 1b).

### 2.5. SRE-Luciferase Activity

The SRE-luciferase assay results paralleled the trends observed in the CRE-luciferase activity. WT ADCY3-expressing cells demonstrated nearly a 3-fold increase in SRE-luciferase activity compared to unstimulated cells, highlighting the strong enhancement of MAPK/ERK pathway signaling by WT ADCY3. Conversely, the SRE-luciferase activity in cells expressing mutant ADCY3 was significantly reduced, indicating a diminished capacity to activate the MAPK/ERK pathway, which is crucial for SRE-driven transcription (Figure 1c).

### 2.6. cAMP Levels

Quantification of intracellular cAMP levels further substantiated the above-mentioned findings. WT ADCY3-expressing cells showed a substantial increase in cAMP levels following forskolin treatment, nearly tripling the levels observed in unstimulated cells. This result underscores the functional efficacy of WT ADCY3 in enhancing cellular cAMP production. However, cells expressing mutant ADCY3 exhibited a modest increase in cAMP levels, much lower than those observed in WT cells, reflecting the mutant ADCY3’s diminished ability to synthesize cAMP (Figure 1d).

These results collectively demonstrate that WT ADCY3 effectively enhances both cAMP production and the activation of downstream transcriptional pathways, whereas the mutant ADCY3 exhibits a significantly impaired functionality in these processes.

### 2.7. Oil Red O Staining of Lipid Accumulation in 3T3-L1 Cells Expressing Wild-Type and Mutant ADCY3

**Figure 2:**
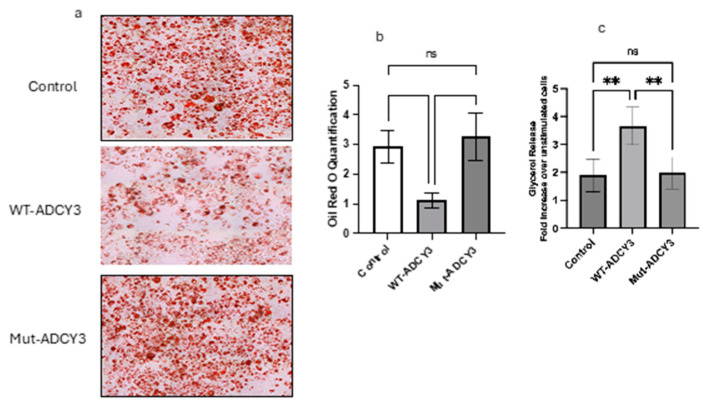
**Lipid accumulation and lipolysis in 3T3-L1 cells expressing wild-type and mutant ADCY3.** (**a**) Oil Red O staining of differentiated 3T3-L1 cells. Control cells (top panel) transfected with an empty vector showed moderate lipid accumulation. Cells expressing wild-type (WT) ADCY3 (middle panel) displayed reduced lipid accumulation, with fewer and smaller lipid droplets. Cells expressing mutant ADCY3 (bottom panel) exhibited increased lipid accumulation, with larger and more densely packed lipid droplets. (**b**) Quantification of lipid accumulation from Oil Red O staining. WT ADCY3-expressing cells had significantly lower lipid content compared to control and mutant ADCY3-expressing cells (Control: 2.92 ± 0.55; WT-ADCY3: 1.130 ± 0.25; Mut-ADCY3: 3.27 ± 0.80; n = 3). (**c**) Lipolysis assay showing glycerol release in forskolin-stimulated 3T3-L1 cells. WT ADCY3-expressing cells exhibited a higher glycerol release compared to mutant ADCY3-expressing cells, indicating enhanced lipolytic activity (Control: 1.9 ± 0.59; WT-ADCY3: 3.66 ± 0.67; Mut-ADCY3: 1.98 ± 0.56; n = 3). Error bars represent the standard deviation from three independent experiments. ** *p* < 0.01. Note: ns: not significant.

To explore the functional differences between wild-type (WT) and mutant ADCY3 on lipid metabolism, 3T3-L1 cells were differentiated into adipocytes and subjected to Oil Red O staining to assess lipid accumulation. Control cells, which were transfected with an empty vector, displayed a moderate level of lipid accumulation, characterized by a consistent distribution of lipid droplets. This provided a baseline measure of adipogenesis under normal conditions, without the influence of ADCY3 overexpression (Figure 2a).

In stark contrast, cells overexpressing WT ADCY3 exhibited significantly lower lipid accumulation compared to control cells. The lipid droplets in WT ADCY3-expressing cells were notably fewer and smaller, suggesting that WT ADCY3 enhances lipid mobilization or promotes more efficient lipid metabolism. This reduction in lipid content is likely due to WT ADCY3’s ability to effectively regulate lipolytic pathways, leading to increased breakdown and turnover of stored lipids. These findings indicate that WT ADCY3 plays a crucial role in maintaining lipid homeostasis within adipocytes, potentially preventing excessive lipid storage by facilitating lipid mobilization and utilization.

On the other hand, cells expressing the mutant form of ADCY3 demonstrated a pronounced increase in lipid accumulation compared to both the control and WT ADCY3-expressing cells. The lipid droplets in these cells were larger and more densely packed, suggesting that the mutation in ADCY3 impairs its function, leading to reduced lipid mobilization. The increase in lipid storage in mutant ADCY3-expressing cells may be due to the loss of normal ADCY3 function, which is critical for regulating lipid metabolism and preventing excessive adipogenesis.

Quantitative analysis of the lipid content supported these visual observations. WT ADCY3-expressing cells had significantly lower lipid accumulation, reinforcing the hypothesis that WT ADCY3 enhances lipid turnover. Conversely, mutant ADCY3-expressing cells exhibited higher lipid content, indicating a failure in the mechanisms that typically regulate lipid mobilization and storage (Figure 2b).

### 2.8. Lipolysis Assay

To further investigate the impact of WT and mutant ADCY3 on lipid metabolism, a lipolysis assay was conducted by measuring glycerol release, a marker of triglyceride breakdown (Figure 2c). Upon stimulation with forskolin, WT ADCY3-expressing cells demonstrated a substantial increase in glycerol release, showing approximately a 3.5-fold enhancement compared to unstimulated controls. This significant rise in glycerol release underscores the role of WT ADCY3 in promoting lipolysis, effectively breaking down stored triglycerides into free fatty acids and glycerol, which are essential for energy production and metabolic regulation.

In contrast, cells expressing mutant ADCY3 exhibited a markedly reduced glycerol release, with only about a 2-fold increase over unstimulated cells. This diminished lipolytic activity suggests that the mutation in ADCY3 disrupts its normal function, leading to impaired triglyceride breakdown. The reduced glycerol release in mutant ADCY3-expressing cells points to a potential defect in the signaling pathways that regulate lipolysis, further contributing to the observed increase in lipid storage.

These results collectively highlight the critical role of WT ADCY3 in regulating lipid metabolism within adipocytes. WT ADCY3 appears to facilitate the efficient breakdown and turnover of lipids, thereby preventing excessive lipid accumulation and promoting metabolic health. In contrast, the loss-of-function mutation in ADCY3 leads to decreased lipolysis and increased lipid storage, which could predispose cells to lipid overload and related metabolic disorders.

### 2.9. In Silico Structural Analysis

The predicted full-length structure of the human ADCY3 (UniProt accession #: 060266) was obtained utilizing the AlphaFold protein structure prediction tool [12] The crystal structure of the adenylyl cyclase 9 (ADCY9) bound to the activated stimulatory G-protein subunit was obtained from the RSCB database (PDB ID: 6R3Q) [13,14]. Structural alignment of ADCY3 and ADCY9 was established for the Cα atoms using Pymol (The PyMOL Molecular Graphics System, Version 2.0.0, Schrödinger, LLC; pymol.org). Protein–protein docking was performed using the HDock web server [14,15].

### 2.10. The ADCY3 Nonsense Mutation Negatively Impacts ADCY3 Binding to the Stimulatory G-Protein Subunit and Subsequent Activation of ADCY3

**Figure 3:**
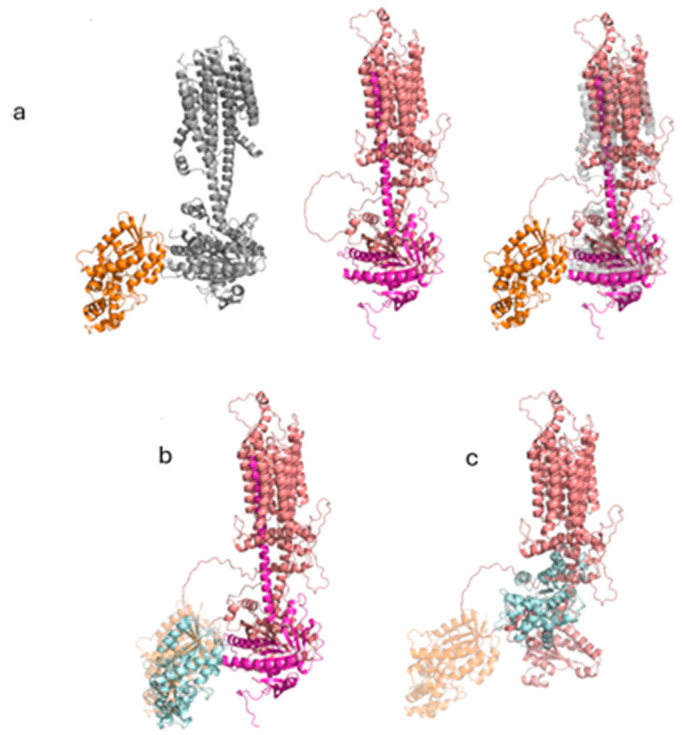
**The truncated segment of ADCY3 is required for its activation by the stimulatory G-protein subunit**. (**a**) Left panel: Cartoon representation of ADCY9 (gray) bound to the activated G-protein subunit (orange). Middle panel: Cartoon representation of WT ADCY3 (salmon) highlighting the truncated segment (magenta). Right panel: Cartoon representation of the aligned ADCY3 (salmon) and ADCY9 (transparent gray) bound to the stimulatory G-protein unit (orange) and highlighting the truncated segment in ADCY3 (magenta). (**b**) Cartoon representation of the highest-score docked stimulatory G-protein subunit (cyan) with the intracellular domain of the WT ADCY3. ADCY3 is colored in salmon, and the truncated region in magenta. The original stimulatory G-protein subunit pose is represented in a transparent orange color. (**c**) Cartoon representation of the highest-score docked stimulatory G-protein subunit (cyan) with the intracellular domain of the truncated ADCY3 (salmon). The original stimulatory G-protein subunit pose is represented in a transparent orange color.

The transmembrane enzyme ADCY3 is activated by the binding of the stimulatory G-protein subunit to its intracellular domain. To investigate whether this interaction is affected by the truncated homozygous variant, we performed in silico structural analysis. Since the crystal structure of ADCY3 is not available in the RCSB database, we obtained the predicted full-length structure of the protein through the AlphaFold protein structure prediction tool (UniProt accession #: 060266) (Figure 3a, middle panel). We then searched the RCSB database for the crystal structure of a closely related adenylyl cyclase enzyme bound to the activated stimulatory G-protein subunit. This search led us to the structure of the activated ADCY9 bound to the stimulatory G-protein subunit (PDB ID: 6R3Q) (Figure 3a, left panel). The structural alignment of the two cyclases revealed great similarities. More importantly, the activated G-protein subunit specifically interacts with the intracellular region that is missing in the truncated cyclase (Figure 3a, right panel).

To confirm how truncation affects this interaction, we performed protein–protein docking between the stimulatory G-protein subunit and the intracellular domain of both the WT and truncated ADCY3 using the HDOCK web server. The results showed a higher docking score for the WT ADCY3 (−317) with a confidence score of 0.9657, indicating stronger binding affinity (Figure 3b) compared to the truncated ADCY3 which had docking and confidence scores of −234 and 0.844, respectively. Importantly, the root-mean-square deviation (RMSD) values from the original G-protein subunit coordinates were 8.212 Å for the WT and 55.735 Å for the truncated docked G-protein subunit. Overall, these results indicate a stronger binding affinity of the stimulatory G-protein subunit to the WT ADCY3 compared to the truncated protein. The truncation also appears to shift the interfacial interaction between the two proteins to a different location, suggesting that the truncated ADCY3 cannot be activated by the stimulatory G-protein subunit (Figure 3c).

## 3. Discussion

In this study, we report a novel homozygous p.Thr840X germline mutation in the *ADCY3* gene in a four-year-old patient, who started to gain weight from early childhood. The *ADCY3* gene has two transmembrane regions and two catalytic domains [16]. The nonsense variant in our patient is located in exon 15 located in a transmembrane region of the *ADCY3* gene. The mutated protein in our patient is predicted to be 840 amino acids instead of 1114 amino acids, leading to a truncated protein. Pathogenic variants in the transmembrane region predominantly lead to loss of the stability of the *ADCY3* receptor [16]. 

The association of the *ADCY3* gene in the development of obesity has been emphasized by several studies from different populations [5,8,9]. Loss-of-function variants in the *ADYC3* gene lead to severe obesity [6], whereas a gain-of-function variant was found to have a protective association against food-induced obesity [7]. Most of the reported homozygous ADCY3 variants (five cases) were reported by Saeed et al. from Pakistan, where two of the reported cases were born to consanguineous parents [5]. Surprisingly, our patient is from Pakistani consanguineous parents. Genetic testing is advisable for consanguineous families as consanguinity increases the likelihood of inheriting pathogenic alleles which may predispose to rare diseases and autosomal recessive diseases such as monogenic childhood obesity.

*ADCY3*, a member of an adenylyl cyclase family, is anchored in the plasma membrane and converts ATP to cAMP. The cAMP signaling is involved in regulating the expression of genes associated with lipolysis, thermogenesis, adipogenesis [17], food intake [18], and leptin sensitivity [19]. To assess the pathogenicity of the novel p.Thr840X variant, we undertook in vitro functional analysis by transfecting 3T3-L1 cells with wild-type and mutant *ADCY3* plasmids and their respective luciferase reporter constructs with forskolin stimulation.

The localization of *ADCY3* is enriched throughout the brain and especially in the primary cilia of the hypothalamic neurons, which suggests its role in energy homeostasis [3]. The generation of cAMP plays a vital role in the regulation of the downstream pathways such as protein kinase-A (PKA), the cyclic AMP response element-binding protein (CREB), and AMP-activated protein kinase (AMPK) [4]. The impaired function in mutant *ADCY3*-expressing cells significantly reduces their ability to activate these crucial transcriptional pathways, which could have downstream effects on cellular metabolism and function.

*ADCY3* is involved in various physiological metabolic pathways such as control of lipid and carbohydrate metabolism, adipose tissue development, and regulating the expression of genes involved in lipolysis, adipogenesis, and thermogenesis [17]. This finding also suggests that the mutant form of ADCY3 impairs the lipolytic process of the loss-of-function mutation in ADCY3 and results in decreased lipolytic activity, leading to higher lipid storage and less glycerol release, highlighting the importance of ADCY3’s functionality in regulating lipid metabolism.

## 4. Materials and Methods

Written consent was obtained from both parents of the patient to participate in this study. The study was approved by the Institutional Review Board (IRB) of Sidra Medicine, Qatar (IRB reference number 1689931). Peripheral blood was collected from the patient and her parents, and genomic DNA was extracted using the QIAamp DNA blood midi kit (Cat. 51185, Qiagen, Hilden, Germany). For next-generation sequencing, exonic and flanking regions of 52 obesity-associated genes (gene panel) were captured using an optimized set of DNA hybridization probes and sequenced using the Illumina NovaSeq 6000 (Illumina, San Diego, CA, USA). For detailed DNA sequencing and analysis methodologies, please refer to our recently published article [20]. 

### 4.1. 3T3-L1 Cell Culture and Differentiation

#### 4.1.1. Cell Culture

3T3-L1 pre-adipocytes were obtained from the American Type Culture Collection (ATCC) and maintained in Dulbecco’s Modified Eagle Medium (DMEM) supplemented with 10% fetal bovine serum (FBS), 1% penicillin-streptomycin, and 1% L-glutamine. Cells were cultured at 37 °C in a humidified atmosphere containing 5% CO2. The culture medium was refreshed every 2–3 days, and cells were passaged when they reached approximately 70–80% confluence using trypsin-EDTA.

#### 4.1.2. Differentiation into Adipocytes

To induce differentiation, 3T3-L1 cells were seeded in 6-well plates at a density of approximately 2 × 104 cells/cm2 and allowed to grow to confluence. Two days post-confluence (designated as day 0), differentiation was initiated by replacing the culture medium with DMEM containing 10% FBS, 1% penicillin-streptomycin, and a differentiation cocktail consisting of 0.5 mM 3-isobutyl-1-methylxanthine (IBMX), 1 µM dexamethasone, and 10 µg/mL insulin. After 48 h (day 2), the differentiation medium was replaced with DMEM containing 10% FBS and 10 µg/mL insulin. Cells were maintained in this insulin-containing medium for an additional 6 days, with medium changes every 2 days. By day 8, the cells had differentiated into mature adipocytes, characterized by the accumulation of lipid droplets, which was confirmed by Oil Red O staining.

### 4.2. Western Blot for ADCY3 Protein

Protein lysates from 3T3-L1 cells transfected with either wild-type (WT) or mutant ADCY3 were prepared using RIPA buffer supplemented with protease inhibitors. Equal amounts of protein (20–30 µg) were loaded onto a 4–12% NuPAGE Bis-Tris gel (Thermo Fisher Scientific, Waltham, MA, USA) and separated by SDS-PAGE electrophoresis. The proteins were then transferred onto a PVDF membrane using a semi-dry transfer system. After blocking with 5% non-fat milk in TBS-T for 1 h at room temperature, the membrane was incubated overnight at 4 °C with a primary antibody against ADCY3 (diluted according to the manufacturer’s recommendation). The membrane was then washed and incubated with an HRP-conjugated secondary antibody for 1 h at room temperature. GAPDH was used as a loading control and was detected by incubating the membrane with a GAPDH-specific primary antibody followed by an HRP-conjugated secondary antibody. Detection was performed using an enhanced chemiluminescence (ECL) substrate, and the bands were visualized using a chemiluminescent imaging system.

### 4.3. Luciferase Assay

Undifferentiated 3T3-L1 pre-adipocytes were seeded in 24-well plates at 5 × 10^4^ cells/well and transfected at 70–80% confluence using Lipofectamine 3000 and plasmids encoding wild-type (WT) or mutant ADCY3, along with a CRE-luciferase or SRE-luciferase reporter plasmid. A *Renilla* luciferase plasmid was co-transfected for normalization. Twenty-four hours post-transfection, cells were treated with forskolin (10 µM) for 4 h to stimulate cAMP production and activation of the CRE or SRE pathways. Following forskolin treatment, cells were lysed in Passive Lysis Buffer (Promega), and firefly and *Renilla* luciferase activities were measured using the Dual-Luciferase^®^ Reporter Assay System (Promega) according to the manufacturer’s instructions. Luminescence was detected using a microplate luminometer. Firefly luciferase activity was normalized to *Renilla* luciferase activity to control for differences in transfection efficiency. The normalized luciferase activity was expressed as fold change over unstimulated control cells.

### 4.4. cAMP Assay

Undifferentiated 3T3-L1 cells were transfected at 70–80% confluence using Lipofectamine 3000 and plasmids encoding either wild-type (WT) or mutant ADCY3, following the manufacturer’s protocol. Twenty-four hours post-transfection, cells were treated with forskolin (10 µM) for four hours to stimulate cAMP production. Following stimulation, cells were lysed using the lysis buffer provided in the cAMP ELISA Kit (Colorimetric) from Cell Biolabs Inc. (San Diego, CA, USA). The cAMP levels were measured according to the manufacturer’s instructions, and absorbance was read at 450 nm using a microplate reader.

### 4.5. WT and Mutant ADCY3 Overexpression and Oil Red O Staining

For Oil Red O staining experiments, 3T3-L1 cells were transfected at 70–80% confluence using Lipofectamine 3000 and plasmids encoding either wild-type (WT) or mutant ADCY3, according to the manufacturer’s instructions (Thermo Fisher Scientific, Waltham, MA, USA). Twenty-four hours post-transfection, cells were subjected to the standard differentiation protocol to induce adipogenesis. On day 8 of differentiation, cells were fixed with 10% formalin for 30 min, rinsed with PBS and 60% isopropanol, and then stained with freshly prepared Oil Red O solution for 15 min. Excess stain was removed with distilled water, and lipid droplets were visualized as bright red puncta. To quantify lipid accumulation, the Oil Red O stain was extracted with 100% isopropanol, and the absorbance was measured at 520 nm using a microplate reader.

### 4.6. Lipolysis Assay Using Differentiated 3T3-L1 Cells

3T3-L1 cells were cultured and differentiated into adipocytes, and on day 8 of differentiation, lipolysis was assessed using Abcam’s Lipolysis Assay Kit (Colorimetric) following the manufacturer’s instructions. Cells were transfected with either wild-type (WT) or mutant ADCY3 plasmids prior to differentiation. For the assay, differentiated adipocytes were washed with PBS and incubated with fresh DMEM containing 10% FBS. Forskolin (10 µM) was added to stimulate lipolysis, with unstimulated cells receiving vehicle (DMSO). After a 4 h incubation at 37 °C, the culture medium was collected, centrifuged, and supernatants were analyzed for glycerol release. Absorbance was measured at 570 nm. Glycerol concentrations were calculated using a standard curve, and results were expressed as fold change relative to unstimulated control cells.

### 4.7. Statistical Analysis

Data represent means ± SD of three independent experiments. Asterisks indicate a significant difference using one-way ANOVA followed by Dunnett’s analysis.

## 5. Conclusions

Our in vitro and in silico functional studies of the novel p.Thr840X variant in the *ADCY3* gene showed impaired receptor activation, reduced cAMP production, and impaired adipogenic and lipolytic activity. Taken together, these findings suggest that the nonsense variant leads to a loss of ADCY3 function, which in turn impairs enzymatic activity and contributes to the development of obesity, consistent with the patient’s phenotype. This underscores the importance of genetic testing in cases of severe early-onset obesity, as early diagnosis may enable personalized treatment strategies that could significantly benefit affected patients.

## Data Availability

All relevant data are included in the manuscript. Further original data will be made available by contacting the corresponding authors within the regulations of the ethical approval.
